# Effects of Novel Mutations in the *LEPR* Gene on Litter Size in Gobi Short Tail Sheep and Sonid Sheep

**DOI:** 10.3390/vetsci12090868

**Published:** 2025-09-06

**Authors:** Sen Yang, Lin An, Pengda Dong, Ming Zhang, Guifang Cao, Taogetao Baoying, Lai Da, Changqing Li, Bin Tong

**Affiliations:** 1 The State Key Laboratory of Reproductive Regulation and Breeding of Grassland Livestock, School of Life Sciences, Inner Mongolia University, Hohhot 010020, China; 15047753359@163.com (S.Y.); iamanlin@outlook.com (L.A.); 13281527895@163.com (P.D.); guifangcao@126.com (G.C.); 2 Inner Mongolia Mengyuan Sheep Breeding Company, Baotou 014016, China; meiligeng777@126.com; 3 Animal Husbandry Center of Sonid Right Banner, Xilingol 026399, China; byn7896@126.com; 4 Inner Mongolia Academy of Agricultural and Animal Husbandry Sciences, Hohhot 010031, China; dalai1023@163.com (L.D.); lcqeg@126.com (C.L.)

**Keywords:** association, *LEPR*, litter size, Gobi short tail sheep, Sonid sheep

## Abstract

Increasing the litter size in sheep is a primary objective in breeding programs, driving sustained interest in identifying candidate functional genes and molecular markers associated with fecundity. The known FecD mutation in the *LEPR* gene has been shown to regulate reproductive traits by influencing the ovulation rate of Davisdale sheep. However, the relationship between single-nucleotide polymorphisms (SNPs) in the *LEPR* and litter size remains unknown in Gobi short tail sheep and Sonid sheep. In this research, we found one novel variant and thirteen known variants through direct sequencing in Sonid sheep and Gobi short tail sheep and performed an association study in a large-scale population. The association analysis identified two SNPs (c.240C>T (rs159694506) and c.279C>T (rs159694508)) with significant litter size associations in Gobi short tail sheep (*p* < 0.01). In Sonid sheep, the c.240C>T (rs159694506), c.279C>T (rs159694508), g.41249772C>T (rs412130067), g.41249873A>C (rs425490800), g.41250357T>C (rs424307284), and g.41250358T>C (rs404651806) SNPs were significantly associated with litter size (*p* < 0.05). In addition, in GB sheep, the frequency of the litter-size-associated C allele at the c.240C>T (rs159694506) and c.279C>T (rs159694508) variants were significantly lower than that in Sonid sheep (SN), Mongolia sheep (MG), Ujimqin sheep (UM), Tan sheep (Tan), Hu sheep (Hu), and Small-tailed Han sheep (STH) populations. In SN sheep, the frequency of the litter-size-associated C allele at the c.240C>T (rs159694506) and c.279C>T (rs159694508) variant was significantly lower than that in the DPU. These findings provided valuable molecular markers pertinent to the fecundity of sheep, offering scientific evidence for the genetic improvement of these breeds.

## Simple Summary

The *LEPR* gene is considered a major genetic factor influencing sheep reproduction. Here, we performed association analyses of 14 *LEPR* variants with litter size in Gobi short-tail and Sonid sheep breeds. Furthermore, we compared the favorable allele frequency of the two variants across Gobi short tail sheep (GB), Sonid sheep (SN), Mongolia sheep (MG), Ujimqin sheep (UM), Dorper × Ujimqin F1 population (DPU), Suffolk × Ujimqin F1 population (SFKU), Tan sheep (Tan), Hu sheep (Hu), and Small-tailed Han sheep (STH). Association results demonstrated that the known c.240C>T (rs159694506) and c.279C>T (rs159694508) variants could influence the litter size in Gobi short tail sheep and Sonid sheep, whereas the known g.41249772C>T (rs412130067), g.41249873A>C (rs425490800), g.41250357T>C (rs424307284), and g.41250358T>C (rs404651806) variants identified herein showed a specific association with the litter size in Sonid sheep. In addition, in GB sheep, the frequency of the litter-size-associated C allele at the c.240C>T (rs159694506) and c.279C>T (rs159694508) variants was significantly lower than that in SN, UM, Tan, Hu, and STH sheep populations. In SN sheep, the frequency of the litter-size-associated C allele at the c.240C>T (rs159694506) and c.279C>T (rs159694508) variants was significantly lower than that in DPU. These data provide potential genetic markers for sheep breeding, and the novel variants also have value for the study of ovine *LEPR*.

## 1. Introduction

Litter size serves as a key indicator of the reproductive capacity of sheep and a critical economic trait in sheep production. Promoting the reproductive performance of mutton sheep can reduce the breeding cost and improve the breeding efficiency. Due to the low fecundity of most sheep breeds, progress in increasing the litter size produced by traditional breeding methods has been slow. However, compared with traditional breeding methods, marker-assisted selection (MAS) enables efficient genetic composition analysis and genotype screening at the molecular level. This methodology facilitates precise selection breeding with the aid of molecular markers, thereby accelerating genetic gains and enhancing breeding efficiency [1]. Therefore, efforts are underway to identify novel single-nucleotide polymorphisms (SNPs) and screen genetic markers associated with reproductive traits, utilizing MAS technology to enhance the efficiency and accuracy of selective breeding, with the aim of potentially increasing the litter size in sheep. Furthermore, many variants identified in reproduction-related genes, growth differentiation factor 9 (*GDF9*), and leptin receptor *(LEPR*), are considered to be factors influencing reproductive traits in sheep, including seasonal estrus, ovulation rate, and litter size [2]. The genetic variants impacting the litter size and ovulation rate have been discovered across numerous ovine breeds. Nevertheless, it is plausible that these variants could be present in populations that have not been thoroughly characterized yet, and there might also be novel mutations that have not been identified.

The process of animal reproduction is inherently complex, influenced by multiple genes and loci associated with reproductive characteristics, such as litter size and ovulation rate. The analysis of the collective impacts of multiple loci on reproductive traits is of significant importance [3,4]. Research has reported on the integrated effects of FecX^L^ (rs3508196091) (impacting *BMP15*) and FecL^L^ (rs588626728) (impacting *B4GALNT2*) on Lacaune sheep [5]. Similarly, the litter size in Small-tailed Han (STH) sheep is collectively influenced by FecB (rs3508196091) (influencing *BMPRIB*) and FecX^G^ (rs3506316216) (influencing *BMP15*) [6]. These findings suggest that diverse genetic mechanisms govern ovulation and reproductive characteristics across different sheep populations.

*LEPR* is a leptin receptor gene, which has an effect on the reproductive traits of sheep [7]. Its expression has been detected at both mRNA and protein levels within follicular cells across various species [8]. The development of sheep oocytes is influenced by leptin through the mitigation of mitochondrial dysfunction and oxidative stress [9]. Remarkably, both leptin and *LEPR* proteins are expressed throughout all developmental stages of goat follicles, intricately participating in the regulation of oocyte maturation and embryonic formation [10]. In addition, low doses of leptin stimulation have been observed to stimulate follicle growth while concurrently reducing reactive oxygen species (ROS) levels, along with expressions of Bcl-2-associated X protein (BAX) and active caspase-3. Conversely, elevated concentrations of leptin may trigger caspase 3 activation through the death receptor pathway, diverging from the mitochondrial pathway, thereby compromising cell viability. These findings underscore leptin’s dual role in ovarian tissue, mitigating follicular atresia while simultaneously modulating ovarian function through its concentration-dependent regulatory mechanisms [11]. During oocyte maturation, leptin facilitates the development of oocytes, as evidenced by accelerated blastocyst formation and reduced cellular apoptosis. Therefore, it exerts a sustained impact on the expression of critical genes involved in early embryonic formation [12]. Interestingly, in in vitro culture studies of sheep follicles, appropriate doses of leptin have been found to promote follicle growth in sheep oocytes [13]. When bound to its receptor, the leptin hormone initiates a cascade of cellular signaling pathways, leading to the modulation of cellular physiological and metabolic activities [14]. Additionally, research indicated that the physiological activities of animal reproduction, spanning puberty, estrus, gestation, nursing period, and even early stages of embryo formation, can be influenced by the *LEPR* gene [15,16]. Meanwhile, three variants in the *LEPR* gene (FecD (rs411478947), rs428867159, and rs405459906) have been found to be associated with decreased ovulation rates and litter size in Davisdale sheep [17]. The known FecD (Fecundity Davisdale) variant in *LEPR* has been shown to regulate reproductive traits by influencing the ovulation rate in Davisdale sheep [18]. Firstly, it is necessary to ascertain whether the reproductive capacity of Gobi short tail (GB) and Sonid (SN) sheep is affected by the three known variants. Secondly, efforts should be made to identify new variations in the *LEPR* in GB sheep and SN sheep with the aim of enhancing the reproductive capacity of sheep populations.

Originating from selective breeding of Mongolia (MG) sheep, the GB sheep—an exemplary breed—predominantly inhabits desert and semi-desert regions across Damao Banner, Dorbod Banner, and Urad Middle Banner in Inner Mongolia, China [19]. The SN sheep, a fat-tailed Mongolian sheep breed, produces high-quality meat and coarse wool. It demonstrates robust adaptation to arid grasslands and deserts across northern China and southern Mongolia, with advantageous body conformation and foraging efficiency [20]. Esteemed as a novel and outstanding breed, the Gobi short tail sheep garners widespread admiration. However, its limited fecundity poses a constraint on the advancement of the Inner Mongolia sheep industry. To date, our laboratory has identified nine novel mutations in the *LEPR* gene across MG and Ujimqin sheep populations [21]. However, the reproductive potential of GB sheep remains poorly characterized. Gobi sheep reproductive potential lacks genetic characterization. Therefore, the primary objectives of this research report encompass the following: (1) Evaluate the individual association of four known *LEPR* variants with litter size in GB and SN sheep. (2) Search for novel variants in the *LEPR* gene in GB and SN sheep. (3) Investigate the associations of these novel variants with prolificacy traits across both GB sheep and SN sheep. This study establishes a theoretical foundation for molecular-marker-assisted breeding to increase the litter size in GB and SN sheep, offering a new perspective on *LEPR* functions in ovine reproductive traits.

## 2. Materials and Methods

### 2.1. Ethics Standards

All animal procedures complied with the Administration of Affairs Concerning Experimental Animals (Ministry of Science and Technology, 2004), China. The experimental protocol was approved by the Institutional Animal Care and Use Ethics Committee of Inner Mongolia University (approval number IMU-2015-03) on 15 May 2015.

### 2.2. Samples and Data

We utilized 231 GB sheep raised at Dorbod Banner Sheep Farm, including 210 that produced a single lamb and 21 that produced twin lambs. The 153 SN sheep sourced from Sonid Right Banner, Xilingol League, Inner Mongolia, China included 116 that produced a single lamb and 37 that produced twin lambs. All ewes were two years old and had records of only their first litter size. The average litter size of GB sheep in the experimental cohort was 1.09 lambs per ewe, whereas that of SN sheep was 1.24. Under comparable conditions, there was no specific ram used for mating. All of the sheep were raised under similar conditions with free access to food and water. All matings occurred naturally. Blood samples, each comprising 2 mL, were obtained from the jugular vein of sheep, processed using EDTA-K2 anticoagulant tubes, and subsequently stored at −20 °C.

### 2.3. DNA Extraction and Sequencing

The DNA utilized in this experiment comprises whole-genomic DNA extracted from the blood samples of the sheep. Ten GB sheep (five ewes that produced twin lambs and five ewes that produced a single lamb) and ten SN sheep (five ewes that produced twin lambs and five ewes that produced a single lamb) were randomly selected for DNA extraction from blood samples. The Tiangen Blood Genomic DNA Extraction Kit (catalog number: DP304; Tiangen Biotechnology Co., Ltd., Beijing, China) was employed for DNA extraction, with operational procedures strictly adhering to the instructions provided. To ensure the integrity of the DNA, it was stored at −20 °C, and its quality and concentration were evaluated using agarose gel electrophoresis and UV spectrophotometry.

We meticulously designed ovine-specific PCR primers using Primer 5.0 software (Premier Bio soft International, Palo Alto, CA, USA); the promoter, exon (the primer design referenced was previously conducted in our laboratory [21]), and 3′ UTR regions of the *LEPR* gene in both GB sheep and SN sheep were separately amplified (NCBI reference sequence: NC_056054.1) (Table S1). The products after PCR amplification were sent to the Beijing Genomics Institute (BGI, Beijing, China) for sequencing.

### 2.4. iPLEX MassARRAY SNP Genotyping

Using the MassARRAY^®^ SNP genotyping system (Agena Bioscience, San Diego, CA, USA), we genotyped the 14 variants (one novel mutation and thirteen known mutations) and the three known variants (FecD (rs411478947), rs428867159, and rs405459906) in 231 GB sheep and 153 SN sheep, respectively. PCR and extension primers for the *LEPR* gene were designed from sequences encompassing each target mutation and approximately 100 upstream and downstream bases, utilizing the Assay Design Suite (http://agenabio.com/assay-design-suite-20-software, accessed on 15 December 2023) with default settings (Table S2). Genotyping of each allele was performed on the MassARRAY iPLEX platform. The resulting data were analyzed with MassARRAY Typer 4.0 Analyzer software (Agena Bioscience, San Diego, CA, USA).

### 2.5. Statistical Analysis

The genotypic and allelic frequencies, as well as the Hardy–Weinberg equilibrium, were calculated for the GB sheep and SN sheep populations consisting of 231 and 153 individuals, respectively. The genetic diversity indices, observed heterozygosity (H_o_), expected heterozygosity (H_e_), effective allele numbers (n_e_), and polymorphism information content (PIC) were computed using Nei’s methods [22]. An assessment of linkage disequilibrium (LD), including D′ and *r*^2^, was performed using HAPLOVIEW v. 4.2 [23]. The significant differences between allelic frequencies of SNPs among species were analyzed using the χ^2^ test [24]. A two-way chi-squared test was used to assess the genetic influences of each SNP in the GB sheep and SN sheep alleles on litter size [21]. Results with values less than ten were excluded from the analysis due to insufficient statistical power resulting from a small sample size. All results were reported as mean ± standard error of the mean (SEM).

## 3. Results

### 3.1. Mutations Discovery in the LEPR Gene of Gobi Short Tail Sheep and Sonid Sheep

**Figure 1:**
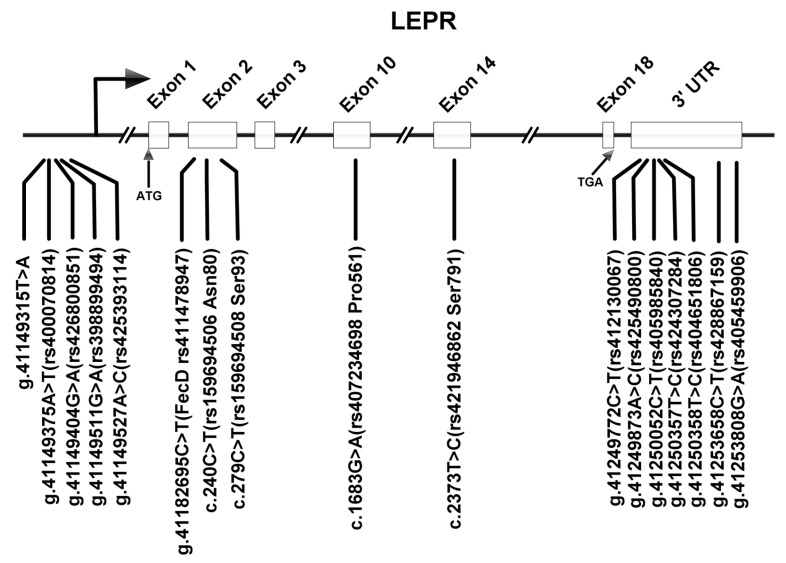
The relative positions of each variant in *LEPR*. These SNPs are located on chromosome 1 of Oar_rambouillet_v2.0 (GenBank accession: NC_056054.1).

A sequence result analysis revealed 14 variants in *LEPR* of GB sheep and SN sheep. The synonymous *LEPR* variants c.240C>T (rs159694506) and c.279C>T (rs159694508) in exon 2, c.1683G>A (rs407234698) in exon 10, and c.2373T>C (rs421946862) in exon 14 matched those previously documented in Mongolia sheep by our laboratory [21]. In this study, we discovered one novel variant within the *LEPR* gene in the GB and SN sheep. Specifically, five single-nucleotide variants (g.41149315T>A, g.41149375A>T (rs400070814), g.41149404G>A (rs426800851), g.41149511G>A (rs398899494), g.41149527A>C (rs425393114)) in the promoter region and five single nucleotide variants (g.41249772C>T (rs412130067), g.41249873A>C (rs425490800), g.41250052C>T (rs405985840), g.41250357T>C (rs424307284), g.41250358T>C (rs404651806)) in the 3′ UTR were identified in the *LEPR* gene. Furthermore, the three previously reported variants identified in Davisdale sheep were not detected in the GB and SN sheep populations; however, we still annotated these three mutations on the gene map, which facilitates a better understanding of their positions within the gene. (Figure 1) The direct sequencing results of each variant are shown in Supplementary Figure S1.

### 3.2. Genetic Diversity Analysis

For each identified variant, frequencies for two alleles and three genotypes, as well as genetic diversity indices (H_o_, H_e_, n_e_, and PIC), were recorded separately for GB and SN sheep (Table 1). In both GB sheep and SN sheep, three genotypes were identified at all 14 variants. A comparison of the wild-type allele frequencies between GB and SN at each variant revealed that the wild-type allele frequency of SN was higher than that of GB at all variants except for the variants g.41149315T>A, c.2373T>C, and g.41250052C>T.

For two SNPs (c.1683G>A, c.2373T>C), the PIC values indicate that low polymorphism is observed in the GB breed (PIC < 0.25). The PIC values of the other 12 SNPs in the GB and SN sheep populations (g.41149315T>A, g.41149375A>T, g.41149404G>A, g.41149511G>A, g.41149527A>C, c.240C>T, c.279C>T, g.41249772C>T, g.41249873A>C, g.41250052C>T, g.41250357T>C, g.41250358T>C) show moderate polymorphism (0.25 < PIC < 0.5), with no highly polymorphic SNP observed (PIC > 0.5).

### 3.3. Linkage Disequilibrium Analysis of Mutations in the LEPR Gene

**Figure 2:**
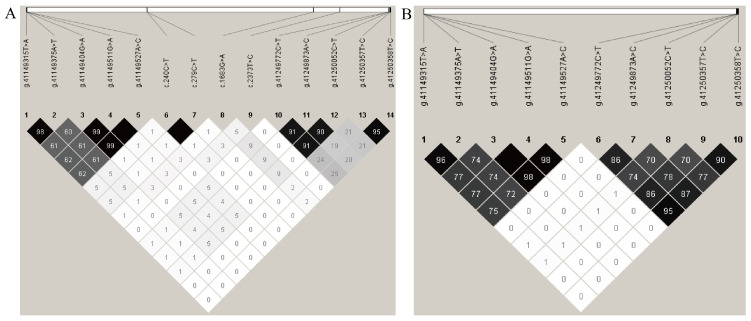
The linkage disequilibrium analysis of each variant in the *LEPR* gene. Numbers represent *r*^2^ × 100. (**A**): Gobi short tail sheep; (**B**): Sonid sheep. Different colors represent the degree of linkage disequilibrium between two variants, with darker colors indicating a higher degree of linkage disequilibrium between the two variants.

To ascertain the linkage relationships among each SNP in GB sheep, estimations of D′ and *r*^2^ were conducted. The results indicated that in the GB sheep population, the *r*^2^ values were found to be closely approaching complete linkage disequilibrium for the following pairs of SNPs: g.41149315T>A and g.41149375A>T (*r*^2^ = 0.980); g.41149404G>A, g.41149511G>A, and g.41149527A>C (*r*^2^ = 0.992); g.41249772C>T, g.41249873A>C, and g.41250052C>T (*r*^2^ = 0.836); g.41250357T>C and g.41250358T>C (*r*^2^ = 0.927). Consequently, these linkage groups of SNPs were collectively analyzed and designated as a single SNP entity, sequentially labeled as LD1-GB, LD2-GB, LD4-GB, and LD5-GB. Additionally, in the GB breed populations, the SNPs c.240C>T and c.279C>T (*r*^2^ = 1.000) and g.41149511G>A and g.41149527A>C (*r*^2^ = 1.000) were observed to be completely in linkage, assigned as LD3-GB and LD6-GB (Figure 2A).

In the SN breed population, the *r*^2^ values were found to be closely approaching complete linkage disequilibrium for the following pairs of SNPs: g.41149315T>A and g.41149375A>T (*r*^2^ = 0.960); g.41149404G>A, g.41149511G>A, and g.41149527A>C (*r*^2^ = 0.983); g.41249772C>T, g.41249873A>C, g.41250357T>C, and g.41250358T>C (*r*^2^ = 0.860). These were subsequently labeled as LD1-SN, LD2-SN, and LD4-SN. Furthermore, within the SN population, the SNPs c.240C>T and c.279C>T (*r*^2^ = 1.000) and g.41149404G>A and g.41149511G>A (*r*^2^ = 1.000) were fully in linkage and designated as LD3-SN and LD5-SN (Figure 2B). The D′ and *r*^2^ values for GB and SN breeds can be found in Tables S3 and S4.

### 3.4. Associations Between SNPs and Litter Size

**Figure 3:**
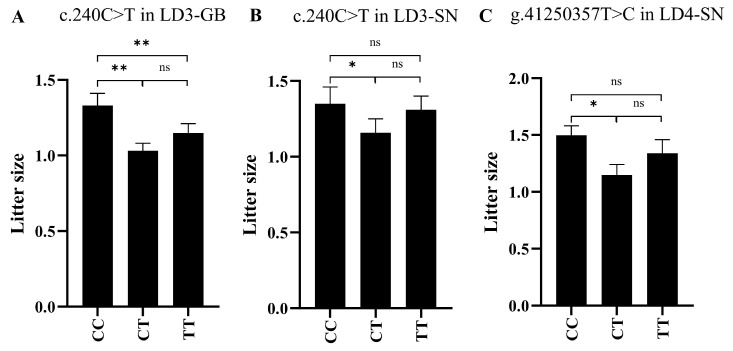
Litter size distribution of a variety of genotypes in different sheep breeds. (**A**) The analysis of the association between different genotypes of c.240C>T in LD3-GB and litter size in GB sheep was conducted. (**B**) The analysis of the association between different genotypes of c.240C>T in LD3-SN and litter size in two breeds of sheep was conducted. (**C**) The analysis of the association between different genotypes of g.41250357T>C in LD4-SN and litter size in two breeds of sheep was conducted. GB: Gobi short tail sheep, SN: Sonid sheep, ns: non-significant; *: *p* < 0.05; **: *p* < 0.01. The SNPs c.240C>T and c.279C>T (*r*^2^ = 1.000) were observed to be completely in linkage, assigned as LD3-GB in Gobi short tail sheep and LD3-SN in Sonid sheep. The SNPs g.41249772C>T, g.41250357T>C, and g.41250358T>C (*r*^2^ = 0.909) were observed to be completely in linkage, assigned as LD4-SN in Sonid sheep.

Due to the impact of the FecB (rs3508196091) variant on the sheep litter size, the FecB testing was initially conducted in GB sheep and SN sheep populations. The results indicated that the FecB variant was not detected in the 384 tested sheep populations. Based on this, the association between the litter size and each SNP in 231 GB sheep and 153 SN sheep was analyzed. The results indicate that in GB sheep, the litter size of the CC genotype of c.240C>T in LD3-GB was significantly higher than that of the CT genotype (0.30 additional lambs, *p* < 0.01) and the TT genotype (0.18 additional lambs, *p* < 0.01). Similarly, in SN sheep, the litter size of the CC genotype of c.240C>T in LD3-SN was significantly higher (0.19 additional lambs, *p* < 0.05) than that of the CT genotypes for g.41250357T>C in LD4-SN, and the litter size of the CC genotype was significantly higher (0.35 additional lambs, *p* < 0.05) than that of the CT genotypes (Figure 3). For more specific information, please refer to Supplementary Tables S5 and S6.

### 3.5. Comparison of the Allele Frequency of Seven Variants in the LEPR Gene

**Figure 4:**
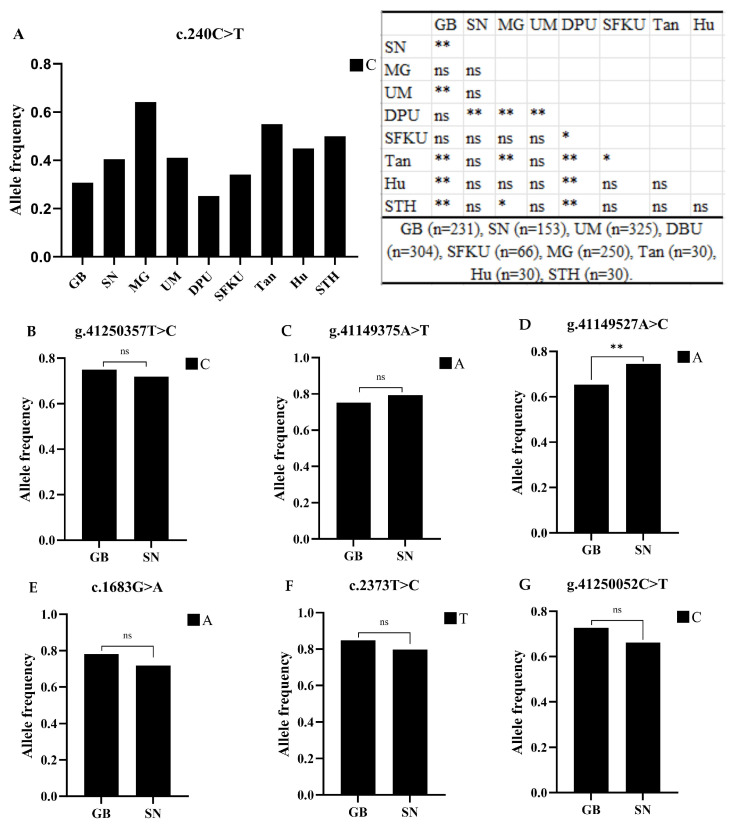
The allele frequency distribution of different variants among different varieties: (**A**) Distribution of the C allele frequency associated with litter size at the c.240C>T variant across nine sheep breeds. (**B**) Distribution of the C allele frequency associated with litter size at the g.41250357T>C variant in GB and SN sheep breeds. (**C**–**G**) Distribution of the major allele frequencies for the g.41149375A>T, g.41149527A>C, c.1683G>A, c.2373T>C, and g.41250052C>T variants in Gobi GB and SN. GB: Gobi short tail sheep, SN: Sonid sheep, MG: Mongolia sheep, UM: Ujimqin sheep, DPU: Dorper × Ujimqin F1 population, SFKU: Suffolk × Ujimqin F1 population, Tan: Tan sheep, Hu: Hu sheep, STH: Small-tailed Han sheep, ns: non-significant; *: *p* < 0.05; **: *p* < 0.01.

The frequencies of dominant alleles of known variants were compared between GB and SN sheep populations, including the litter-size-associated C allele of the g.41250357T>C in LD4-SN variants. In addition, using data previously published by our laboratory, the frequencies of the litter-size-associated C allele at the c.240C>T variants were compared among GB, SN, MG, UM, DPU, SFKU, Tan, Hu, and STH sheep populations [21,25]. The results showed that, in GB sheep, the frequency of the litter-size-associated C allele at the c.240C>T in the LD3-GB variant was significantly lower than that in SN, UM, Tan, Hu, and STH sheep populations. In SN sheep, the frequency of the litter-size-associated C allele at the c.240C>T in the LD3-SN variant was significantly lower than that in DPU, while the other inter-population differences are presented in Figure 4A. Moreover, no significant difference was observed between GB and SN sheep in the frequency of the litter-size-associated C allele at the g.41250357T>C in the LD4-SN variant (Figure 4B). However, when comparing the frequencies of dominant alleles of other variants between GB and SN sheep, it was found that the frequency of the dominant A allele at the g.41149527A>C variant was significantly higher in the SN sheep than in GB sheep, while no significant differences were detected for the other variants (Figure 4C–G).

## 4. Discussion

Previous research has reported a relationship between the FecD variant and the ovulation rate and litter size in Davisdale sheep [26]. Reports also suggested that, as the primary gene influencing the ovulation rate and placental–fetal survival rate, FecD plays a pivotal role [27]. Fourteen variants were detected within the GB sheep population in this study. Although four variants occurred in the exon regions of *LEPR*, codon degeneracy prevented amino acid changes at these positions. Nonetheless, prior evidence indicates that such variants decrease the mRNA minimum free energy (MFE), thereby increasing the *MTHFR* transcript stability and potentially altering its gene expression [28]. Additionally, several silent variants reduced the mRNA stability and translation efficiency, significantly impairing dopamine-stimulated upregulation of *DRD2* expression [29]. Therefore, it can be inferred that c.240C>T and c.279C>T are associated with the litter size, potentially due to the effects of synonymous variants at the RNA and protein levels. Further experimental validation is required to confirm this hypothesis.

Interestingly, research has shown that linkage disequilibrium between SNPs may also impact the litter size in sheep [30]. As the conclusions of this research demonstrate, three SNPs associated with litter size were discovered and were found to be in two linkage disequilibrium groups: LD4-SN (g.41249772C>T, g.41250357T>C, and g.41250358T>C) (*r*^2^ = 0.909) and LD3-GB (c.240C>T and c.279C>T) (*r*^2^ = 1.000). Therefore, it can be inferred that the g.41249772C>T and g.41250358T>C in LD4-SN may also be connected to the litter size in SN sheep. However, the lack of a significant impact on litter size may be attributed to differences in genotypes among individual sheep or to the smaller size of the sheep population. Conversely, both SNPs in LD3-GB were linked to litter size in GB sheep. This suggests that one SNP likely represents the major variant influencing prolificacy in this breed, while other SNPs in LD3-GB may exert potential effects on litter size through linkage disequilibrium. This will necessitate further comprehensive and rigorous research in the future to elucidate its mechanism of action.

Our prior research had identified associations between *GDF9* SNPs (g.46544883A>G, c.1040T>C, and c.46547859C>T) as well as *BMPR1B* variants (g.29346567A>T and c.1470G>T) with litter size in MG sheep [2,24]. Additionally, the current research being conducted has indicated a correlation between *LEPR* variants (g.41250357T>C, c.240C>T, and c.279C>T) and litter size in GB and SN breeds. The GB sheep and SN sheep populations also belong to Mongolia sheep [19], showing a high genetic diversity of reproductive control in the Mongolia sheep system. Thereby, it is hypothesized that a combination of various genes may genetically control the litter size in GB, SN, and other Mongolia sheep populations, with each gene exerting a modest influence, as observed in the Romanov sheep [31]. Notably, the frequencies of the C allele at the c.240C>T and c.279C>T variants were relatively low in both GB and SN sheep populations. Although the litter-size-associated C allele was more common in Hu and MG sheep, its frequency remained lower in GB, SN, DPU, and SFKU sheep. Our findings revealed a clear pattern: at both variants, the frequency of the litter-size-associated C allele was consistently lower in GB sheep. Furthermore, we demonstrated that in both GB and SN populations, individuals carrying the CC genotype at the c.240C>T and c.279C>T variants had a higher average litter size compared with those carrying the CT or TT genotypes. These results suggest that, for future breeding programs, sheep with the CC genotype at the c.240C>T and c.279C>T variants should be prioritized. By contrast, the g.41250357T>C variant in LD4-SN was associated with litter size only in the SN population, indicating its potential as a molecular marker that warrants further validation in larger sample sizes. Overall, the molecular markers identified in this study provide valuable tools for rapidly improving litter size in GB and SN sheep, as well as in other breeds with relatively low reproductive performance.

## 5. Conclusions

In conclusion, the variants c.240C>T (rs159694506) and c.279C>T (rs159694508) are associated with significant litter size in GB and SN sheep populations. The g.41249772C>T (rs412130067), g.41249873A>C (rs425490800), g.41250357T>C (rs424307284), and g.41250358T>C (rs404651806) with significant litter size associations in SN sheep. Furthermore, we confirmed that in GB sheep, the frequency of the litter-size-associated C allele at the c.240C>T variant in LD3-GB was significantly lower than that in SN, UM, Tan, Hu, and STH sheep populations. In SN sheep, the frequency of the litter-size-associated C allele at the c.240C>T variant in LD3-SN was significantly lower than that in DPU. These findings provide valuable molecular genetic markers for promoting the reproductive performance in Mongolia sheep populations. Additionally, they offer new potential avenues for investigating the functionality of the *LEPR* gene.

## Figures and Tables

**Table 1:** Genotypic, allelic frequencies, and diversity parameters of 14 SNPs in the Gobi short-tail sheep and Sonid sheep populations.

Variant	Breed	Genotype Frequency			Allele Frequency		H_o_	H_e_	n_e_	PIC	χ^2^ (HWE)
g.41149315T>A		AA	AT	TT	A	T					
	GB	0.584	0.333	0.082	0.751	0.249	0.626	0.374	1.597	0.304	2.721
	UM	0.627	0.333	0.039	0.794	0.206	0.673	0.327	1.486	0.274	0.058
g.41149375A>T		AA	AT	TT	A	T					
	GB	0.584	0.338	0.078	0.753	0.247	0.628	0.372	1.592	0.303	1.940
	UM	0.627	0.346	0.026	0.801	0.199	0.681	0.319	1.469	0.268	1.110
g.41149404 G>A		GG	GA	AA	G	A					
	GB	0.435	0.439	0.126	0.654	0.346	0.548	0.452	1.826	0.350	0.197
	UM	0.546	0.401	0.053	0.747	0.253	0.622	0.378	1.608	0.307	0.564
g.41149511G>A		GG	GA	AA	G	A					
	GB	0.433	0.442	0.126	0.654	0.346	0.547	0.453	1.827	0.350	0.142
	UM	0.546	0.401	0.053	0.747	0.253	0.622	0.378	1.608	0.307	0.564
g.41149527A>C		CC	CA	AA	C	A					
	GB	0.126	0.442	0.433	0.346	0.654	0.547	0.453	1.827	0.350	0.142
	UM	0.052	0.405	0.542	0.255	0.745	0.620	0.380	1.613	0.308	0.683
c.240C>T		CC	CT	TT	T	C					
	GB	0.078	0.459	0.463	0.307	0.693	0.574	0.426	1.741	0.335	1.396
	UM	0.157	0.497	0.346	0.405	0.595	0.518	0.482	1.931	0.366	0.142
c.279C>T		CC	CT	TT	C	T					
	GB	0.078	0.459	0.463	0.307	0.693	0.574	0.426	1.741	0.335	1.396
	UM	0.157	0.497	0.346	0.405	0.595	0.518	0.482	1.931	0.366	0.142
c.1683G>A		GG	GA	AA	G	A					
	GB	0.035	0.368	0.597	0.219	0.781	0.035	0.368	0.597	0.219	0.781
	UM	0.072	0.418	0.510	0.281	0.719	0.072	0.418	0.510	0.281	0.719
c.2373T>C		TT	CT	CC	T	C					
	GB	0.719	0.260	0.022	0.848	0.152	0.743	0.257	1.346	0.225	0.024
	UM	0.634	0.327	0.039	0.797	0.203	0.677	0.323	1.477	0.271	0.020
g.41249772C>T		CC	CT	TT	C	T					
	GB	0.052	0.416	0.532	0.260	0.740	0.615	0.385	1.625	0.311	1.504
	UM	0.099	0.391	0.510	0.295	0.705	0.584	0.416	1.711	0.329	0.545
g.41249873A>C		CC	CA	AA	C	A					
	GB	0.545	0.402	0.054	0.746	0.254	0.621	0.379	1.611	0.307	0.778
	UM	0.520	0.378	0.101	0.709	0.291	0.588	0.412	1.701	0.328	0.999
g.41250052C>T		CC	CT	TT	C	T					
	GB	0.512	0.429	0.059	0.727	0.273	0.603	0.397	1.659	0.318	1.346
	UM	0.441	0.441	0.118	0.662	0.338	0.552	0.448	1.810	0.347	0.029
g.41250357T>C		TT	CT	CC	T	C					
	GB	0.048	0.407	0.545	0.251	0.749	0.624	0.376	1.603	0.305	1.554
	UM	0.098	0.366	0.536	0.281	0.719	0.596	0.404	1.678	0.322	1.360
g.41250358T>C		TT	CT	CC	T	C					
	GB	0.052	0.416	0.532	0.260	0.740	0.615	0.385	1.625	0.311	1.504
	UM	0.105	0.392	0.503	0.301	0.699	0.579	0.421	1.726	0.332	0.696

Note: GB: Gobi short tail sheep; SN: Sonid sheep; H_o_: observed heterozygosity; H_e_: expected heterozygosity; n_e_: effective allele numbers; PIC: polymorphism information content; HWE: Hardy–Weinberg equilibrium.

## Data Availability

Data are contained within the article and Supplementary Materials.

## Supplementary Materials

The following supporting information can be downloaded at https://www.mdpi.com/article/10.3390/vetsci12090868/s1: Figure S1. The direct sequencing results of each variant in the *LEPR* gene; Table S1. PCR primers used for sequencing of *LEPR*; Table S2. MassARRAY primers used for genotyping 15 variants in *LEPR* gene; Table S3. Linkage disequilibrium as measured using D′ and *r^2^* among variants in Gobi short tail sheep; Table S4. Linkage disequilibrium as measured using D′ and *r^2^* among variants in Sonid sheep; Table S5. The effects of the seven *LEPR* variants on litter size in the Gobi short tail sheep; Table S6. The effects of the seven *LEPR* variants on litter size in the Sonid sheep.
