# Supplementary material for: Effects of Novel Mutations in the LEPR Gene on Litter Size in Gobi Short Tail Sheep and Sonid Sheep
Source: Vet Sci. 2025 Sep 6;12(9):868. doi: 10.3390/vetsci12090868 (PMC12474046; doi:10.3390/vetsci12090868)
Supplement: Supplementary file 1 [file vetsci-12-00868-s001.zip › Figure S1. The direct sequencing results of each variant in the LEPR gene. .pdf]

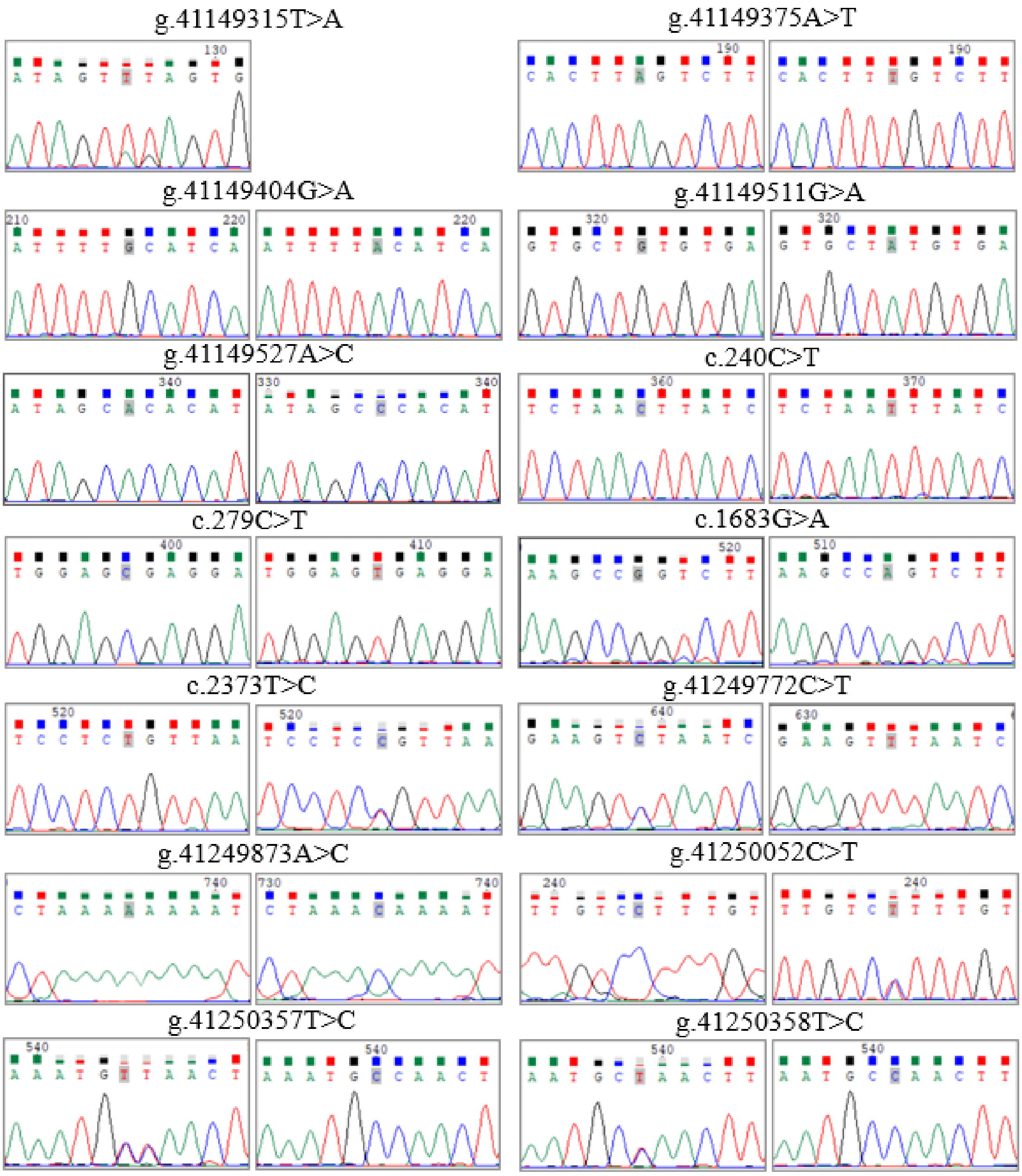

Figure S1. The direct sequencing results of each variant in the *LEPR* gene. These SNPs are located on chromosome 1 of Oar_rambouillet_v2.0 (GenBank accession: NC_056054.1).
