# Supplementary material for: Effects of Novel Mutations in the LEPR Gene on Litter Size in Gobi Short Tail Sheep and Sonid Sheep
Source: Vet Sci. 2025 Sep 6;12(9):868. doi: 10.3390/vetsci12090868 (PMC12474046; doi:10.3390/vetsci12090868)
Supplement: Supplementary file 1 [file vetsci-12-00868-s001.zip › Table S1. PCR primers used for sequencing of LEPR.pdf]

Table S1. PCR primers used for sequencing of *LEPR.*

Name	Target Region	Primer Sequence (5'-3')	Annealing Temperature (°C)	Product Length (bp)
LEPR-1	Promoter	F: CCTGCATTGACTTCTTTACCA<br>R: AAAATGCTGCTGTGAATGAAC	60	740 (740 bp promoter)
LEPR-2	Promoter	F: TACTGACCACTTAAGACCCAT<br>R: CTCTGGATCGTCCCAATGC	60	722 (722 bp promoter)
LEPR-3	Promoter	F: GCTTTCAGTTCATCTACC<br>R: CTTCTTTCATTCCAATCT	60	721 (608 bp promoter + 113 bp 5' UTR)
*LEPR-4**	Exon 1	F: CTGTACATATTTAATTGGCAT<br>R: ATACTCTCCCAAATACACA	52	938 (383 bp 5' UTR + 40 bp exon1 + 515 bp intron 1)
*LEPR-5**	Exon 2	F: CGCAAATAAATGTACCCTC<br>R: TAATATTTGCTCAGAATCACA	60	646 (198 bp intron 1+ 330 bp exon 2 + 118 bp intron 2)
*LEPR-6**	Exon 3	F: TTTTGGGGTCTTCTGTCCTAG<br>R: TTCTCCCTTTCTTCAGTCA	60	700 (281 bp intron 2 + 124 bp exon 3 + 295 bp intron 3)
*LEPR-7**	Exon 4	F: TGTCATTTCTTTGTGTGGAACC<br>R: CCCCCAGCTCCTAGATCAGT	60	1080 (478 bp intron 3 + 209 bp exon 4 + 393 bp intron 4)
*LEPR-8**	Exon 5	F: TTCCAAGTAGTGCTCTGCAAAA<br>R: GGAGAGAAAGGGGGAGAAAA	60	895 (376 bp intron 4 + 146 bp exon 5 + 373 bp intron 5)
*LEPR-9**	Exon 6	F: TTGCAGGCAGCTTCTTAACC<br>R: TTGAAAAGCAAGCATCTGGA	60	985 (411 bp intron 5 + 145 bp exon 6 + 429 bp intron 6)
LEPR-10/11*	Exons 7/8	F: AATGAGAATGCTTGGGATGC<br>R: TCACCAATTTATTTAACTCCTCAA	58	917 (187 bp intron 6 + 291 bp exon 7 + 160 bp intron 7 + 118 bp exon 8 + 161 bp intron 8)
LEPR-12*	Exon 9	F: CCTTCCTTTCCATCTCAGGA<br>R: TGCTTCCTACCCAGCCTTAC	60	1006 (377 bp intron 8 + 200 bp exon 9 + 429 bp intron 9)

LEPR-13*	Exon 10	F: GCAGAGTGACATGGACAATGA<br>R: CCTTGCGTCACTATATGTCCCT	60	1020 (459 bp intron 9 + 159 bp exon 10 + 402 bp intron 10)
LEPR-14/15*	Exons 11/12	F: GAGGGTGATTGGGAAGTGAG<br>R: AACATACGATTGGGCTGGAT	60	927 (262 bp intron 10 + 160 bp exon 11 + 117 bp intron 11 + 83 bp exon 12 + 305 bp intron 12)
LEPR-16*	Exon 13	F: GCTCTTTCTCATTGCTGCTTT<br>R: TTTCAAATCTAAGCAATTACTTGTACC	56	1105 (427 bp intron 12 + 217 bp exon 13 + 461 bp intron 13)
LEPR-17*	Exon 14	F: TGAAGTGGGACTTTGTGCAG<br>R: TGCTGCCAAATTGTTTTCC	60	998 (391 bp intron 13 + 183 bp exon 14 + 424 bp intron 14)
*LEPR-18**	Exon 15	F: AATATTGCTGGTTTTGATCTG<br>R: GAACCATATACCCTTAACACA	60	659 (400 bp intron 14 + 96 bp exon 15 + 163 bp intron 15)
LEPR-19/20*	Exons 16/17	F: ACGATCAAAGCTTTCTGATTTTG<br>R: AAGTTTTGAACCTTGAAAATGTTTG	54	1272 (143 bp intron 15 + 103 bp exon 16 + 851 bp intron 16 + 76 bp exon 17 + 99 bp intron 17)
LEPR-21*	Exon 18	F: CATGTTCTCCTCCCTAATTGTGT<br>R: TCAGTCCAGTTCAGTTTGGATG	60	877 (637 bp intron 17 + 18 bp exon 18 + 222 bp 3' UTR)
LEPR-22	3' UTR	F: TCACTTCCGATGAATCCAA<br>R: GATATGTCAGGTCATCCCA	60	661 (661 bp 3' UTR)

Note: F: forward primer sequence, R: reverse primer sequence, and UTR: untranslated region. *: The primer design of *LEPR* previously published from our laboratory [23].
