# Supplementary material for: Effects of Novel Mutations in the LEPR Gene on Litter Size in Gobi Short Tail Sheep and Sonid Sheep
Source: Vet Sci. 2025 Sep 6;12(9):868. doi: 10.3390/vetsci12090868 (PMC12474046; doi:10.3390/vetsci12090868)
Supplement: Supplementary file 1 [file vetsci-12-00868-s001.zip › Table S2. MassARRAY primers used for genotyping 15 variants in LEPR gene.pdf]

Table S2. MassARRAY primers used for genotyping 15 variants in *LEPR* gene

Name	Target Region	Primer Sequence (5'-3')	Annealing Temperature (°C)
g.41149315T>A	Promoter	F: ACGTTGGATGGATACTTCATCATTTACAGCC	56.9
		R: ACGTTGGATGTGGCCTATAAACCAAGTCTC	
		E: CCTCCCACAACACTT	
g.41149375A>T	Promoter	F: ACGTTGGATGTGGCCTATAAACCAAGTCTC	60.6
		R: ACGTTGGATGGATACTTCATCATTTACAGCC	
		E: CGGCCTGGTTCTAACACTA	
g.41149404G>A	Promoter	F: ACGTTGGATGTGGCCTATAAACCAAGTCTC	53.6
		R: ACGTTGGATGGATACTTCATCATTTACAGCC	
		E: ATTCTCATAGTTAAACTGATG	
g.41149511G>A	Promoter	F: ACGTTGGATGATCCCATTACCCTCATGCAG	49.7
		R: ACGTTGGATGCCTGGTGATTGGTACATGTG	
		E: CCGCGAACAAAGTGCTTTGTGCT	
g.41149527A>C	Promoter	F: ACGTTGGATGCTTTAACATTGAGCCACCTG	50.9
		R: ACGTTGGATGCCCTCATGCAGAGAACAAAG	
		E: TGATTGGTACATGTG	
c.240C>T	Exon 2	F: ACGTTGGATGGGCAGTTGTTGAAACTAAGC	46.6
		R: ACGTTGGATGGTCATCTGCATATACAGAGC	
		E: TCAAGTGGTAACTACTTATCTAA	
c.279C>T	Exon 2	F: ACGTTGGATGGGCAGTTGTTGAAACTAAGC	46
		R: ACGTTGGATGGTCATCTGCATATACAGAGC	
		E: CACTGTTGCTTTTGGAG	

c.1683G>A	Exon 10	F: ACGTTGGATGCCATAGCGAATCTGGAACTG<br>R: ACGTTGGATGTCCATCCAGTGTGAAAGCAG<br>E: CTGAAGATTATTCTCTGGAAAGAC	49.2
c.2373T>C	Exon 14	F: ACGTTGGATGTGCTGTCACCCAGTGATTAC<br>R: ACGTTGGATGACAGTAAGCTTACCATGGAC<br>E: GGCTTAGAATCCCTTCCTC	49.6
g.41249772C>T	3' UTR	F: ACGTTGGATGCTGGCAGATTGGATTCATCG<br>R: ACGTTGGATGTATCTGAACAGAGAACGGAC<br>E: TGAATCGGAAGTGATCATGATTA	58.5
g.41249873A>C	3' UTR	F: ACGTTGGATGAGTTCTCAATAGTTTCACC<br>R: ACGTTGGATGCTTCCCAACATTCTGTAGAG<br>E: GGTCAATAGTTTCACCAATTTT	55.6
g.41250052C>T	3' UTR	F: ACGTTGGATGCAGGTCATCTCAAAAGTAG<br>R: ACGTTGGATGATGTGCTGAGAACAACAGTC<br>E: TGGCTTTTGATTTGTC	54
g.41250357T>C	3' UTR	F: ACGTTGGATGCTAACTCTGATCCATATGGG<br>R: ACGTTGGATGGGCCTTTGACCAGAACAAAC<br>E: GCCTTGAAAATGGCCTAAATG	50.7
g.41250358T>C	3' UTR	F: ACGTTGGATGGGCCTTTGACCAGAACAAAC<br>R: ACGTTGGATGCTAACTCTGATCCATATGGG<br>E: CCCCCTTTGAACTGAAATCAAAGTT	45.3
FecB	Exon 8	F: ACGTTGGATGCCAAGATGTTTTCATGCCTC<br>R: ACGTTGGATGTTCTTCACTACAGAGGAGGC<br>E: CCTCATCAACACCGTC	46.7

Note: F: forward primer sequence, R: reverse primer sequence, E: extended primer sequence.
