# Supplementary material for: Effects of Novel Mutations in the LEPR Gene on Litter Size in Gobi Short Tail Sheep and Sonid Sheep
Source: Vet Sci. 2025 Sep 6;12(9):868. doi: 10.3390/vetsci12090868 (PMC12474046; doi:10.3390/vetsci12090868)
Supplement: Supplementary file 1 [file vetsci-12-00868-s001.zip › Table S3. Linkage disequilibrium as measured using D' and r2 among variants in Gobi short tail sheep.pdf]

Table S3. Linkage disequilibrium as measured using D' and r^2 among variants in Gobi short tail sheep

Breeds<br>SNPs	g.41149315<br>T>A	g.4114937<br>5A>T	g.41149404<br>G>A	g.41149511<br>G>A	g.41149527<br>A>C	c.240<br>C>T	c.279C>T	c.1683G>A	c.2373T>C	g.41249772<br>C>T	g.41249873<br>A>C	g.41250052<br>C>T	g.4125035<br>7T>C
g.41149375<br>A>T	D' = 1.000<br>r^2 = 0.988												
g.41149404<br>G>A	D' = 0.985<br>r^2 = 0.613	D' = 0.985<br>r^2 = 0.606											
g.41149511<br>G>A	D' = 1.000<br>r^2 = 0.626	D' = 1.000<br>r^2 = 0.618	D' = 1.000<br>r^2 = 0.990										
g.41149527<br>A>C	D' = 1.000<br>r^2 = 0.626	D' = 1.000<br>r^2 = 0.618	D' = 1.000<br>r^2 = 0.990	D' = 1.000<br>r^2 = 1.000									
c.240C>T	D' = 0.259<br>r^2 = 0.050	D' = 0.263<br>r^2 = 0.051	D' = 0.132<br>r^2 = 0.015	D' = 0.139<br>r^2 = 0.016	D' = 0.139<br>r^2 = 0.016								
c.279C>T	D' = 0.259<br>r^2 = 0.050	D' = 0.263<br>r^2 = 0.051	D' = 0.132<br>r^2 = 0.015	D' = 0.139<br>r^2 = 0.016	D' = 0.139<br>r^2 = 0.016	D' = 1.000<br>r^2 = 1.000							
c.1683G>A	D' = 0.126<br>r^2 = 0.013	D' = 0.131<br>r^2 = 0.015	D' = 0.246<br>r^2 = 0.032	D' = 0.240<br>r^2 = 0.030	D' = 0.240<br>r^2 = 0.030	D' = 0.125<br>r^2 = 0.010	D' = 0.125<br>r^2 = 0.010						
c.2373T>C	D' = 0.119<br>r^2 = 0.008	D' = 0.112<br>r^2 = 0.007	D' = 0.038<br>r^2 = 0.000	D' = 0.016<br>r^2 = 0.000	D' = 0.016<br>r^2 = 0.000	D' = 0.306<br>r^2 = 0.038	D' = 0.306<br>r^2 = 0.038	D' = 1.000<br>r^2 = 0.050					
g.41249772<br>C>T	D' = 0.135<br>r^2 = 0.017	D' = 0.135<br>r^2 = 0.017	D' = 0.281<br>r^2 = 0.053	D' = 0.275<br>r^2 = 0.050	D' = 0.275<br>r^2 = 0.050	D' = 0.145<br>r^2 = 0.003	D' = 0.145<br>r^2 = 0.003	D' = 1.000<br>r^2 = 0.098	D' = 0.023<br>r^2 = 0.000				
g.41249873<br>A>C	D' = 0.120<br>r^2 = 0.014	D' = 0.118<br>r^2 = 0.014	D' = 0.275<br>r^2 = 0.047	D' = 0.269<br>r^2 = 0.045	D' = 0.269<br>r^2 = 0.045	D' = 0.124<br>r^2 = 0.002	D' = 0.124<br>r^2 = 0.002	D' = 1.000<br>r^2 = 0.092	D' = 0.010<br>r^2 = 0.000	D' = 0.988<br>r^2 = 0.911			

g.41250052	D' = 0.133	D' = 0.132	D' = 0.299	D' = 0.294	D' = 0.294	D' = 0.117	D' = 0.117	D' = 1.000	D' = 0.001	D' = 1.000	D' = 0.964		
C>T	r^2 = 0.017	r^2 = 0.017	r^2 = 0.054	r^2 = 0.052	r^2 = 0.052	r^2 = 0.002	r^2 = 0.002	r^2 = 0.090	r^2 = 0.000	r^2 = 0.912	r^2 = 0.908		
g.41250357T	D' = 0.024	D' = 0.020	D' = 0.043	D' = 0.036	D' = 0.036	D' = 0.040	D' = 0.040	D' = 0.527	D' = 0.071	D' = 0.510	D' = 0.452	D' = 0.470	
>C	r^2 = 0.001	r^2 = 0.000	r^2 = 0.001	r^2 = 0.001	r^2 = 0.001	r2 = 0.001	r^2 = 0.001	r^2 = 0.026	r^2 = 0.003	r^2 = 0.249	r^2 = 0.199	r^2 = 0.211	
g.41250358T	D' = 0.014	D' = 0.009	D' = 0.045	D' = 0.038	D' = 0.038	D' = 0.032	D' = 0.032	D' = 0.460	D' = 0.049	D' = 0.503	D' = 0.468	D' = 0.487	D' = 1.000
>C	r^2 = 0.000	r^2 = 0.000	r^2 = 0.001	r^2 = 0.001	r^2 = 0.001	r^2 = 0.001	r^2 = 0.001	r^2 = 0.021	r^2 = 0.001	r^2 = 0.253	r^2 = 0.204	r^2 = 0.216	r^2 = 0.955
