# Supplementary material for: Effects of Novel Mutations in the LEPR Gene on Litter Size in Gobi Short Tail Sheep and Sonid Sheep
Source: Vet Sci. 2025 Sep 6;12(9):868. doi: 10.3390/vetsci12090868 (PMC12474046; doi:10.3390/vetsci12090868)
Supplement: Supplementary file 1 [file vetsci-12-00868-s001.zip › Table S4. Linkage disequilibrium as measured using D' and r2 among variants in Sonid sheep.pdf]

Table S4. Linkage disequilibrium as measured using D' and r^2 among variants in Sonid sheep

Breeds<br>SNPs	g.41149315<br>T>A	g.41149375<br>A>T	g.41149404<br>G>A	g.41149511<br>G>A	g.41149527<br>A>C	c.240C>T	c.279C>T	c.1683G>A	c.2373T>C	g.41249772<br>C>T	g.4124987<br>3A>C	g.41250052<br>C>T	g.41250357<br>T>C
g.41149375<br>A>T	D' = 1.000<br>r^2 = 0.960												
g.41149404<br>G>A	D' = 1.000<br>r^2 = 0.771	D' = 1.000<br>r^2 = 0.740											
g.41149511<br>G>A	D' = 1.000<br>r^2 = 0.771	D' = 1.000<br>r^2 = 0.740	D' = 1.000<br>r^2 = 1.000										
g.41149527<br>A>C	D' = 1.000<br>r^2 = 0.758	D' = 1.000<br>r^2 = 0.728	D' = 1.000<br>r^2 = 0.983	D' = 1.000<br>r^2 = 0.983									
c.240C>T	D' = 0.147<br>r^2 = 0.008	D' = 0.177<br>r^2 = 0.011	D' = 0.129<br>r^2 = 0.008	D' = 0.129<br>r^2 = 0.008	D' = 0.135<br>r^2 = 0.009								
c.279C>T	D' = 0.147<br>r^2 = 0.008	D' = 0.177<br>r^2 = 0.011	D' = 0.129<br>r^2 = 0.008	D' = 0.129<br>r^2 = 0.008	D' = 0.135<br>r^2 = 0.009	D' = 1.000<br>r^2 = 1.000							
c.1683G>A	D' = 0.171<br>r^2 = 0.019	D' = 0.190<br>r^2 = 0.023	D' = 0.033<br>r^2 = 0.001	D' = 0.033<br>r^2 = 0.001	D' = 0.042<br>r^2 = 0.002	D' = 0.108<br>r^2 = 0.003	D' = 0.108<br>r^2 = 0.003						
c.2373T>C	D' = 0.315<br>r^2 = 0.007	D' = 0.375<br>r^2 = 0.009	D' = 0.208<br>r^2 = 0.004	D' = 0.208<br>r^2 = 0.004	D' = 0.231<br>r^2 = 0.005	D' = 0.202<br>r^2 = 0.015	D' = 0.202<br>r^2 = 0.015	D' = 0.792<br>r^2 = 0.062					
g.41249772<br>C>T	D' = 0.084<br>r^2 = 0.004	D' = 0.068<br>r^2 = 0.003	D' = 0.068<br>r^2 = 0.004	D' = 0.068<br>r^2 = 0.004	D' = 0.055<br>r^2 = 0.003	D' = 0.246<br>r^2 = 0.037	D' = 0.246<br>r^2 = 0.037	D' = 0.446<br>r^2 = 0.032	D' = 0.295<br>r^2 = 0.054				
g.41249873<br>A>C	D' = 0.130<br>r^2 = 0.011	D' = 0.117<br>r^2 = 0.009	D' = 0.091<br>r^2 = 0.007	D' = 0.091<br>r^2 = 0.007	D' = 0.079<br>r^2 = 0.005	D' = 0.262<br>r^2 = 0.040	D' = 0.262<br>r^2 = 0.040	D' = 0.251<br>r^2 = 0.010	D' = 0.245<br>r^2 = 0.039	D' = 0.950<br>r^2 = 0.860			

g.41250052	D' = 0.181	D' = 0.175	D' = 0.147	D' = 0.147	D' = 0.157	D' = 0.162	D' = 0.162	D' = 0.346	D' = 0.300	D' = 1.000	D' = 1.000		
C>T	r^2 = 0.015	r^2 = 0.014	r^2 = 0.013	r^2 = 0.013	r^2 = 0.015	r^2 = 0.021	r^2 = 0.021	r^2 = 0.026	r^2 = 0.041	r^2 = 0.741	r^2 = 0.707		
g.41250357	D' = 0.022	D' = 0.074	D' = 0.013	D' = 0.013	D' = 0.003	D' = 0.228	D' = 0.228	D' = 0.411	D' = 0.306	D' = 0.950	D' = 0.884	D' = 1.000	
T>C	r^2 = 0.000	r^2 = 0.001	r^2 = 0.000	r^2 = 0.000	r^2 = 0.000	r2 = 0.030	r^2 = 0.030	r^2 = 0.026	r^2 = 0.061	r^2 = 0.860	r^2 = 0.782	r^2 = 0.707	
g.41250358	D' = 0.102	D' = 0.088	D' = 0.077	D' = 0.077	D' = 0.087	D' = 0.251	D' = 0.251	D' = 0.334	D' = 0.276	D' = 1.000	D' = 0.983	D' = 1.000	D' = 1.000
T>C	r^2 = 0.006	r^2 = 0.004	r^2 = 0.005	r^2 = 0.005	r^2 = 0.006	r^2 = 0.040	r^2 = 0.040	r^2 = 0.019	r^2 = 0.045	r^2 = 0.954	r^2 = 0.879	r^2 = 0.777	r^2 = 0.909
