# Supplementary material for: Effects of Novel Mutations in the LEPR Gene on Litter Size in Gobi Short Tail Sheep and Sonid Sheep
Source: Vet Sci. 2025 Sep 6;12(9):868. doi: 10.3390/vetsci12090868 (PMC12474046; doi:10.3390/vetsci12090868)
Supplement: Supplementary file 1 [file vetsci-12-00868-s001.zip › Table S5. The effects of the seven LEPR variants on litter size in the Gobi short tail sheep.pdf]

Table S5. The effects of the seven *LEPR* variants on litter size in the Gobi short tail sheep.

Variant	Genotype	Number	Litter Size
g.41149375A>T in LD1-GB	AA	135	1.06 ± 0.05
	AT	78	1.13 ± 0.05
	TT	18	1.32 ± 0.08
g.41149527A>C in LD2-GB	CC	29	1.22 ± 0.06
	CA	102	1.12 ± 0.04
	AA	100	1.05 ± 0.06
c.240C>T in LD3-GB	CC	18	1.33 ± 0.08^{A}
	CT	106	1.03 ± 0.05^{B}
	TT	107	1.15 ± 0.06^{B}
c.1683G>A	GA	85	1.00 ± 0.10
	AA	138	1.20 ± 0.05
c.2373T>C	TT	166	1.04 ± 0.04
	TC	60	1.20 ± 0.05
g.41250052C>T in LD4-GB	CC	131	1.04 ± 0.05
	CT	88	1.19 ± 0.05
	TT	12	1.14 ± 0.09
g.41250357T>C in LD5-GB	TT	11	1.00 ± 0.10
	TC	94	1.18 ± 0.05
	CC	126	1.06 ± 0.05

Note: A, B: $p < 0.01$.
