# Supplementary material for: Effects of Novel Mutations in the LEPR Gene on Litter Size in Gobi Short Tail Sheep and Sonid Sheep
Source: Vet Sci. 2025 Sep 6;12(9):868. doi: 10.3390/vetsci12090868 (PMC12474046; doi:10.3390/vetsci12090868)
Supplement: Supplementary file 1 [file vetsci-12-00868-s001.zip › Table S6. The effects of the seven LEPR variants on litter size in the Sonid sheep.pdf]

Table S6. The effects of the seven *LEPR* variants on litter size in the Sonid sheep.

Variant	Genotype	Number	Litter Size
g.41149375A>T in LD1-SN	AA	96	1.22 ± 0.08
	AT	53	1.38 ± 0.09
g.41149527A>C in LD2-SN	CA	62	1.34 ± 0.08
	AA	83	1.18 ± 0.08
c.240C>T in LD3-SN	CC	24	1.35 ± 0.11[a]
	CT	76	1.16 ± 0.09[b]
	TT	53	1.31 ± 0.09[ab]
c.1683G>A	GG	11	1.20 ± 0.16
	GA	64	1.32 ± 0.09
	AA	78	1.20 ± 0.08
c.2373T>C	TT	97	1.34 ± 0.07
	TC	50	1.32 ± 0.08
g.41250052C>T	CC	60	1.43 ± 0.10
	CT	77	1.32 ± 0.07
	TT	16	1.31 ± 0.12
g.41250357T>C in LD4-SN	TT	15	1.34 ± 0.12[ab]
	TC	56	1.15 ± 0.09[a]
	CC	82	1.50 ± 0.08[b]

Note: a, b: $p < 0.05$.
